# The metabolic fingerprint of Scots pine—root and needle metabolites show different patterns in dying trees

**DOI:** 10.1093/treephys/tpae036

**Published:** 2024-03-25

**Authors:** Stefan Hunziker, Tatiana Nazarova, Michel Kather, Martin Hartmann, Ivano Brunner, Marcus Schaub, Andreas Rigling, Christian Hug, Leonie Schönbeck, Arun K Bose, Bernd Kammerer, Arthur Gessler

**Affiliations:** Forest Dynamics, Swiss Federal Research Institute WSL, Birmensdorf 8903, Switzerland; Forest Dynamics, Swiss Federal Research Institute WSL, Birmensdorf 8903, Switzerland; Core Facility Metabolomics, Albert-Ludwigs-University Freiburg, Freiburg 79014, Germany; Department of Environmental Systems Science, Institute of Agricultural Sciences, ETH Zurich, Zurich 8092, Switzerland; Forest Soils and Biogeochemistry, Swiss Federal Research Institute WSL, Birmensdorf 8903, Switzerland; Forest Dynamics, Swiss Federal Research Institute WSL, Birmensdorf 8903, Switzerland; Forest Dynamics, Swiss Federal Research Institute WSL, Birmensdorf 8903, Switzerland; Department of Environmental Systems Science, Institute of Terrestrial Ecosystems, ETH Zurich, Zurich 8092, Switzerland; Forest Dynamics, Swiss Federal Research Institute WSL, Birmensdorf 8903, Switzerland; Forest Dynamics, Swiss Federal Research Institute WSL, Birmensdorf 8903, Switzerland; Department of Botany and Plant Sciences, University of California, Riverside, CA 9252, USA; Forest Dynamics, Swiss Federal Research Institute WSL, Birmensdorf 8903, Switzerland; Forestry and Wood Technology Discipline, Khulna University, Khulna 9208, Bangladesh; Core Facility Metabolomics, Albert-Ludwigs-University Freiburg, Freiburg 79014, Germany; Forest Dynamics, Swiss Federal Research Institute WSL, Birmensdorf 8903, Switzerland; Department of Environmental Systems Science, Institute of Terrestrial Ecosystems, ETH Zurich, Zurich 8092, Switzerland

**Keywords:** antioxidants, defense, defoliation, fine roots, GC–MS, osmoprotection

## Abstract

The loss of leaves and needles in tree crowns and tree mortality are increasing worldwide, mostly as a result of more frequent and severe drought stress. Scots pine (*Pinus sylvestris* L.) is a tree species that is strongly affected by these developments in many regions of Europe and Asia. So far, changes in metabolic pathways and metabolite profiles in needles and roots on the trajectory toward mortality are unknown, although they could contribute to a better understanding of the mortality mechanisms. Therefore, we linked long-term observations of canopy defoliation and tree mortality with the characterization of the primary metabolite profile in needles and fine roots of Scots pines from a forest site in the Swiss Rhone valley. Our results show that Scots pines are able to maintain metabolic homeostasis in needles over a wide range of canopy defoliation levels. However, there is a metabolic tipping point at around 80–85% needle loss. Above this threshold, many stress-related metabolites (particularly osmoprotectants, defense compounds and antioxidants) increase in the needles, whereas they decrease in the fine roots. If this defoliation tipping point is exceeded, the trees are very likely to die within a few years. The different patterns between needles and roots indicate that mainly belowground carbon starvation impairs key functions for tree survival and suggest that this is an important factor explaining the increasing mortality of Scots pines.

## Introduction

Climate change is negatively affecting the functioning of forest ecosystems and is increasing tree mortality (Allen et al. 2010; Hartmann et al. 2018). In particular, hot droughts that reduce soil water availability and increase atmospheric evaporative demand cause dieback of forests globally (Allen et al. 2015). In Europe, especially, drought-sensitive Norway spruce shows strong signs of vitality loss and mortality mainly as a consequence of hot and dry summers such as in 2018 (Schuldt et al. 2020). In addition, Scots pine, a species with a large area of distribution all over Eurasia that has been thought to be more drought tolerant, has been subjected to drought-induced mortality since the 1990s (Bigler et al. 2006; Hereş et al. 2012; Etzold et al. 2019).

On a mechanistic basis, mortality following drought is assumed to be mainly caused by hydraulic failure, carbon starvation (Mcdowell et al. 2008; Adams et al. 2017) and as contributing factor infestation by pests and diseases (Oliva et al. 2014), all of which are mutually inclusive (Hajek et al. 2022). Hydraulic failure refers to the disruption of water transport through xylem embolism as well as to cellular desiccation, causing cessation of biochemical functioning (McDowell 2011). Carbon starvation occurs when the supply from photosynthesis and storage can no longer meet the carbon demand for the maintenance of the cellular and defensive metabolism (Mcdowell et al. 2008). It has also been shown that carbon allocation to sink tissues such as roots is negatively affected by drought (Hagedorn et al. 2016; Joseph et al. 2020) and reduced carbon availability in general (Huang et al. 2019), and thus organ-specific carbon starvation might also occur especially in roots, while leaves might be further supplied with assimilates.

Under stressful conditions, the acclimation potential of trees depends inter alia on the availability of assimilates to be converted to osmoprotectants (which can compensate for low water availability on a cellular level), scavengers for reactive oxygen species (that can mitigate damage to membranes and other cellular constituents) and defense compounds and their precursors (to avoid additional biotic damage) (Alexou et al. 2007; De Diego et al. 2013; Jansen et al. 2014; Du et al. 2015; Huang et al. 2020). Thus, not only quantitative carbon availability but also the activity of particular metabolic pathways might be decisive for a tree to cope with stress and thus for its survival or mortality. MacAllister et al. (2019) observed, for example, an increase in osmoprotectants and defense compounds in needles of Scots pine seedlings during a mortality-inducing drought. In Douglas-fir seedlings, atmospheric heat and drought increased the polyol levels acting as osmoprotectants as well as the products of the shikimic acid pathway related to plant defense and antioxidant activity in needles (Jansen et al. 2014).

Vitality of trees is often monitored visually by assessing crown defoliation, which can be done quickly and with a reasonable amount of resources repeatedly and spatially systematically under field conditions (Eichhorn et al. 2016). Moreover, crown defoliation is an excellent predictor for tree mortality (Dobbertin and Brang 2001), and Hunziker et al. (2022) showed that Scots pine trees that reached a crown defoliation of ≥75% as a result of drought died within 1 (70%) or 2 years (83%) without being exposed to further drought stress. Schönbeck et al. (2018) found that soluble sugars and starch concentrations in roots and needles were not affected by an irrigation treatment that released pine trees from drought (compared with the naturally drought exposed controls) but that defoliation and thus reduced needle area had an impact. In particular, starch concentrations in roots were strongly reduced in trees with high defoliation.

Our study was performed at the Pfynwald research site in the canton of Valais in Switzerland, where Scots pine is growing at the dry edge of its area of distribution and is widely subjected to drought-induced needle loss and mortality (Bose et al. 2022). In this region, drought is the central and direct driver of mortality for Scots pine, and it has been shown that hot and dry summers are clearly related to increased defoliation and mortality (Hunziker et al. 2022). At this site, we quantified the mortality risk for trees with different degrees of defoliation.

To better understand the relationship between drought-affected tree vitality (i.e., defoliation as an indicator of mortality risk) and metabolic responses, we assessed the root and needle primary metabolic profile in Scots pine of different defoliation classes. We applied a metabolic fingerprinting approach meant to assess the metabolite profiles for hydrophilic and semipolar compounds following a robust standard procedure (Fiehn et al. 2000; Fiehn 2006). Metabolic fingerprinting stands in-between classical targeted metabolite analysis (where only few, a priori defined metabolites are aimed to be quantified with maximal accuracy) and cutting-edge metabolomics (where the aim is to identify as many compounds as possible from the primary and secondary metabolism). With the method applied, between ~50 and 300 metabolites (depending on plant species and tissue) can be identified, allowing assessment of the central changes in the primary metabolism (e.g., Fiehn et al. 2000; Jansen et al. 2014).

We hypothesized that (i) increased defoliation would be a good predictor for mortality in Scots pine at our research site. In addition, we assumed that increasing defoliation would continually increase the stress-related metabolites (defense, antioxidants and osmoprotectants) in needles as an acclimation response to counteract decreased tree vitality. As an alternative hypothesis, we might assume that the mainly primary metabolites we assess here are kept constant over large defoliation ranges until a metabolic tipping point is reached. We also hypothesized that even at very high defoliation (and thus preceding mortality), when carbon availability is assumed to be strongly reduced, the needle levels of stress-related (and compensating) metabolites remain increased. (ii) The roots will be supported less than the needles and a strong reduction of most metabolites (including the stress-related ones) is hypothesized to occur at high defoliation, indicating organ-specific carbon starvation.

Our study aims to shed light on processes leading to Scots pine mortality on the level of primary metabolites and metabolic pathways.

## Materials and methods

### Study site and plant material

The Pfynwald study site is located in the Rhone Valley in Switzerland (46° 18′ N, 7° 3′ E, 615 m above sea level). The area is one of the driest inner Alpine valleys of the European Alps, with a mean annual temperature of 10.6 °C (19.6 °C for June–August) and mean annual precipitation of 576 mm (174 mm for June–August) for the period of 1995–2014 (data from the MeteoSwiss station Sion) (MeteoSwiss 2018). The soil is shallow and is characterized by low soil volumetric water content that ranges from 0.11 to 0.47 with an average of 0.27 (based on the data from 2003 to 2014) and by a low available water-holding capacity of 135 mm until 0.8 m rooting depth (Bose et al. 2022). The overstory of the forest is dominated by even-aged and more than 100-year-old Scots pines. The mean canopy height reaches 12 m, and the stem density is ca 730 stems per ha. The site is close to the dry edge of distribution of Scots pine, and Scots pine mortality events occur in the whole region since the 1990s (Bigler et al. 2006; Hunziker et al. 2022). Pubescent oak (*Quercus pubescens* Willd.) and shrub species occupy 60% of the understory cover (Brunner et al. 2009; Dobbertin et al. 2010). The research site consists of eight plots, four of which are naturally dry and four have been irrigated since 2003. For this study, we only used naturally dry trees without irrigation (for a detailed description of the Pfynwald experiment, see Bose et al. 2022). The site is located in a forest reserve and, thus, unmanaged.

Defoliation (synonym: crown transparency) was measured once a year (starting in 2003) in ~270 Scots pine trees on the (non-irrigated) control sites. Crown transparency assessment was performed as described by Dobbertin et al. (2004) by visual rating of the crown defoliation in 5% steps using reference photographs ranging from 0% (= a fully foliated tree) to 100% (= a fully defoliated and dead tree). In contrast to the general rounding up or down to the next 5% defoliation step, a value of 95% is attributed to nearly completed defoliated trees as soon as some living needle tissue can be identified. A 100% defoliated tree (if still standing) was re-assessed in the following year to confirm mortality.

On the 4 July 2018, we selected 15 Scots pine individuals on the Pfynwald monitoring site according to their defoliation (determined approximately a month before sampling).

We binned the trees to five different defoliation classes (1 = 0–22% needle loss, 2 = 23–42% needle loss, 3 = 43–62%, 4 = 63–82%, 5 = 83–95% needle loss; similar to Schönbeck et al. 2018) with three replicates per class. To avoid the inclusion of trees in the tissue sampling not maintaining basic vital tree functions anymore, it was assured before taking the samples that trees of the highest defoliation class 5 still carried at least 5% of green needles.

For the defoliation classes 1 and 5, three trees were uprooted, each with a help of a tractor. An ~25-m long rope was attached around the tree trunk at breast height and was connected to the tractor. The rope was first carefully and then increasingly loaded to uproot the tree without splitting the trunk. This procedure was chosen to be able to identify fine roots belonging to a particular tree, which is almost impossible by excavating roots from the soil as the rhizospheres of neighboring trees overlap (Gao et al. 2021). For each of the uprooted trees, three fine root (<2 mm in diameter) samples were taken at different positions of the root plate. Roots were immediately washed with demineralized water to remove the adhering soil particles and were dried again with paper tissue. In addition, three needle samples from the current year cohort were taken from different positions in the upper sunlight part of the crown directly after uprooting. Only green needles without any visual signs of damage or discoloration were collected. Root and needle samples were immediately frozen in liquid nitrogen for quenching and stored at −80 °C back in the laboratory.

For trees of the defoliation classes 2–4, current year needles were collected from the standing trees (and not uprooted trees) on the next day either with extendable loppers or from scaffolds reaching up to the canopy. For the trees of the classes 1 and 5, three samples from different parts of the upper sun-exposed crown (same criteria for the needles collected as given above) were taken and were immediately quenched in liquid nitrogen to rapidly stop the metabolic activity. All plant materials were collected between 10:00 and 14:00 CET.

### Metabolite profiling

Metabolite analysis was performed with GC–MS according to Jansen et al. (2014).

Hydrophilic and semipolar low-molecular-weight metabolites were extracted from fine roots and needles and were derivatized according to a modified method from Erxleben et al. (2012). For each sample, ~10–20 mg of homogenized frozen tissue powder was weighed into a pre-frozen 2-mL round-bottom Eppendorf tube, and for each mg of sample, 50 μL of extraction medium cooled to −80 °C was added (90 vol.% methanol, 10 vol.% ultrapurified (Milli-Q) water and 1 μg mL^−1^ Phenyl-β-D-glucopyranoside as an internal standard for quality control and internal normalization). Table S1 available as Supplementary data at *Tree Physiology* Online shows that the fresh weight/dry weight ratios of needles and roots did not differ among defoliation classes, and thus no bias due to potentially changing tissue water contents was introduced when basing the metabolite abundance on fresh weight. The 50 μL (needles) or 250 μL aliquots (roots) of supernatant were dried under vacuum in 1.5-mL microfuge tubes after centrifugation. Dried extracts were methoximated by adding 20 μL of a 20 mg mL^−1^ solution of methoxyamine hydrochloride in anhydrous pyridine and were incubated at 28 °C for 90 min with shaking at 1400 RPM. For trimethylsilylation, 70 μL of *N*-methyl-*N*-(trimethylsilyl) trifluoroacetamide (Sigma) was transferred to each tube and was incubated at 37 °C for 30 min with 1400 RPM shaking. An *n*-alkane retention index (RI) calibration mixture was created by adding 10 μL of *n*-alkane mix [*n*-Alkane-Mix 16 (C10-C40 even), Cat.-No.:14640, concentration: 50 μg mL^−1^ in *n*-hexane; Neochema, Germany] to 40 μL of *n*-hexane. After a short vortex, reaction mixtures were centrifuged at 14,000 × *g*, 20 °C for 2 min and then 30 μL of supernatant were transferred to amber GC–MS vials with low volume inserts and screw top seals (Agilent Technologies, Palo Alto, CA, USA) for GC–MS analysis. Additionally, 30 μL of each sample was pooled into one quality control sample which was separated into 30-μL aliquots, which were used for column equilibration as well as quality controls.

Derivatized metabolite samples were analyzed on an Agilent GC/MSD system comprised of an Agilent GC 7890A gas chromatograph (Agilent Technologies) fitted with a GERSTEL MultiPurpose Sampler (MPS2-XL, GERSTEL, Mülheim, Germany) and 5975C Inert XL EI/CI MSD quadrupole MS detector (Agilent Technologies). The capillary column used was HP-5MS 5% Phenyl Methyl Silox, length: 60 m, diameter: 0.25 mm, film thickness: 0.25 μm (Agilent Technologies). The GC–MS run conditions were set up according to Erxleben et al. (2012) with some slight adaptations. The GC column oven was held at the initial temperature of 80 °C for 3 min and then at 325 °C for 5 °C min^−1^ before being held at 325 °C for 14 min. Total run time was 66 min. Inlet temperature was 230 °C, transfer line temperature was 280 °C and the MS source temperature was 230 °C. Samples were injected in a randomized order with *n*-hexane blank as the first and *n*-alkane standard as the second injection. These samples were followed by seven consecutive injections of separate aliquots of the pooled sample. Then, after each sixth sample injection, a pooled sample was injected as a quality control.

The raw data files were processed with the free automated mass spectral deconvolution and identification system (version 2.69) software supplied by National Institute of Standards and Technology (NIST, Gaithersburg, MD, USA). After peak filtering and deconvolution, Kováts’ RIs were calculated from the *n*-alkane standard residence times and applied to all samples. A compound matrix was generated in the SpectConnect software, where spectral mass features were matched and grouped.

The compounds were then identified by comparison to the NIST spectral library based on RI and mass spectra similarities of the fragments. For identification, the deviation of the RI had to be <5% and the spectral similarity should be >75%.

The relative quantification of the identified metabolites peaks was achieved by calculating the area of the ion signal and normalizing to the internal standard. Metabolites detected not in all replicates for a given defoliation class were discarded for the comparison between defoliation classes. There were no qualitative changes in the metabolite composition as only one component (maltose) was not present in all defoliation classes.

A critical step in the derivatization process that can negatively affect derivatization efficiency is the silylating step, which is particularly sensitive to moisture. Problems in this step can be detected through the occurrence and abundance of polysiloxanes (Fiehn 2006). These were monitored for all samples, and no indications for any derivatization problems were detected. Moreover, since all ion signals were normalized to the also derivatized internal standard, potentially occurring slight differences in derivatization efficiency were corrected for.

For completeness and to better characterize carbohydrate changes, we included the starch values determined by Schönbeck et al. (2018) for the same defoliation classes at the same site.

### Data analysis and statistics

#### Mortality

The mortality rates (fraction of dying trees) of the single tree defoliation classes were calculated for the time period of 2003–21. In a first step, the inventory data were quality controlled and erroneous or unplausible data points and time series were removed. For each time step in the defoliation time series, it was checked whether the tree was still alive or dead at the time of the inventory in the following year. The cases of trees that were dead were, in the following year, summed up within each defoliation class and were divided by the total number of observations in the respective defoliation class. Multiplying this proportion of dying trees by 100 gave the percentage mortality rate. The same procedure was repeated to examine mortality after 2, 3, 4, 5 and 6 years. The significance of differences in mortality frequency between defoliation classes was tested (separately for mortality within 1–6 years) using a Pearson’s chi-square test, followed by a pairwise comparison for proportions using the Bonferroni method to adjust the *P*-value (prop.test and pairwise.prop.test in the R Development Core Team base package, 2022).

#### Metabolites

For a general comparison among defoliation classes, the differences of the corrected and normalized areas for the specific metabolites were calculated as log ratios of the defoliation classes 2–5 versus the least defoliated class 1. Results were displayed in a heatmap, and cluster analysis (group average and Euclidian distance) was performed to group metabolites and defoliation classes (Heatmap app in OriginPro 2016; OriginLab, Northampton, MA, USA). Moreover, for each metabolite, we performed linear regression to detect the statistical differences (*P* < 0.05) in the needles and roots between the defoliation classes. For this, we considered the defoliation class 1 (0–22% needle loss) as the reference and compared this class with the other defoliation classes for each of the metabolites. The model summary of the linear regression provides the statistics (estimate, standard error and *P*-value) for each of those comparisons. The statistical tests were performed using the programming language R (R Development Core Team 2022), and the ‘lm’ function of the base package of R was used to execute the linear regression.

Absolute normalized peak areas for metabolites were used in a principal component analysis (PCA) using all metabolites present in all replicates of at least one defoliation class in a correlation matrix (OriginPro 2016; OriginLab). For a detailed comparison of changes in antioxidant, defense and osmoprotection-related metabolites in the roots and needles between trees of defoliation classes 1 and 5, we performed, for each metabolite, Student’s *t*-test (class 1 vs class 5; NCCS 2020, NCSS, LLC. Kaysville, UT, USA), selected the metabolites that showed significant differences at *P* < 0.05 at least in one of the organs (roots or needles) and calculated the log ratio changes.

## Results

### Defoliation and mortality

**Figure 1:**
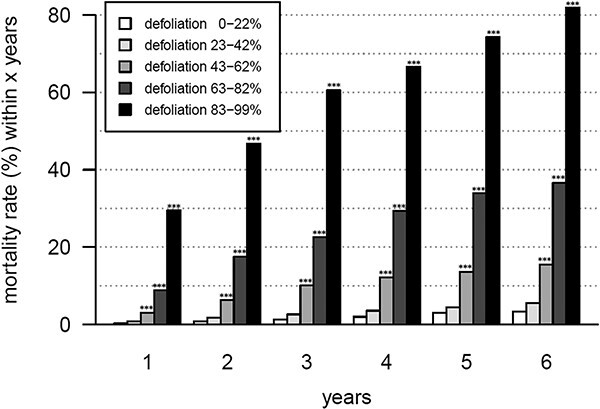
Mortality rates of Scots pines in the different defoliation classes (0–22% = class 1, 23–42% = class 2, 3 = 43–62%, 4 = 63–82%, 83–99% = class 5) within 1–6 years. Asterisks indicate significantly different mortality rates of the classes 2–5 from class 1 (^*^^*^^*^: *P* < 0.001, ^*^^*^: 0.001 < *P* < 0.010, ^*^: 0.010 < *P* < 0.050). The period analyzed includes the years from 2003 to 2021.

Most of the Scots pine trees at the Pfynwald site were highly defoliated before mortality; 29% of the trees from the defoliation class 5 (83–99% defoliation) died within 1 year (Figure 1), 47% died within 2 years and 61% died within 3 years. After 6 years, most of these trees were dead (84%). The probability to die within a few years was, thus, several times higher in defoliation class 5 compared with the other defoliation classes. Already, in the second highest defoliation class 4 (63–82% defoliation), the mortality rate within 6 years was much lower (37%). Most of the trees from other defoliation classes subject to later mortality pass to defoliation class 5 on their trajectory to tree death. After falling into class 5, the trees hardly recovered in the longer term and usually survived for a few years at most. The mortality rates differed significantly between the different defoliation groups (*P* < 0.001). The pairwise comparison of mortality rates showed no significant differences between defoliation classes 1 and 2, but there were highly significant differences between all other combinations of defoliation classes (*P* < 0.001).

Increased defoliation was also significantly associated with decreases in the increment of the diameter at breast height and shoot growth (Table S1 available as Supplementary data at *Tree Physiology* Online). However, needle carbon isotope composition (δ^13^C), which is a measure for intrinsic water-use efficiency (Farquhar et al. 1982), and nitrogen content were not affected. Moreover, there was no significant difference between defoliation classes 1 and 5 in the needle phosphorus content (Table S1 available as Supplementary data at *Tree Physiology* Online).

### Metabolic profiles in needles across the five defoliation classes

**Figure 2:**
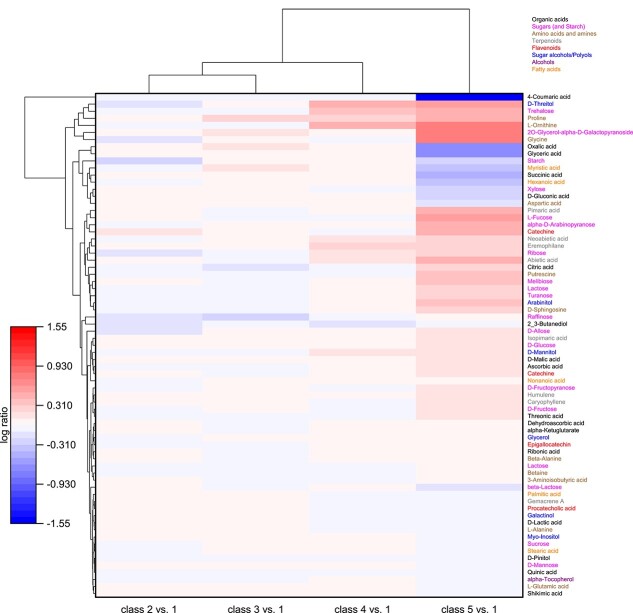
Heatmap of log ratio changes of the metabolite abundances of Scots pine needles across different defoliation classes at the Pfynwald site. Defoliation classes are: 1 = 0–22% needle loss, 2 = 23–42% needle loss, 3 = 43–62%, 4 = 63–82%, 5 = 83–95% needle loss; *n* = 3 (with 3 technical replicates per tree). Blue color indicates a decrease and red color an increase in abundance compared to class 1. Cluster analysis shows Euclidean distance.

The cluster analysis based on the log ratio changes in the defoliation classes 2–5 as related to class 1 (0–22%; Figure 1) shows some dissimilarities in the metabolite abundances found in the needles of the different defoliation classes (Figure 2).

In general, the strongest differences were found between class 5 and the other four classes. While some compounds such as the amino acid proline showed a rather continuous change across defoliation classes, some metabolites (e.g., D-threitol, trehalose, ornithine, ribose and mannitol) started to increase in class 4, but the majority of changes was observed in the needles of the highly defoliated trees (83–95% defoliation) of class 5 (e.g., 4-coumaric acid, glycine, oxalic acid, succinic acid, melibiose, malic acid, turanose and arabinitol; for statistics, see Table S2 available as Supplementary data at *Tree Physiology* Online).

The metabolite showing the strongest decrease in class 5 was 4-coumaric acid (0.001 < *P* < 0.010; Table S2 available as Supplementary data at *Tree Physiology* Online). A cluster of compounds with strong increases in class 5 (and partially in class 4) comprised D-threitol, trehalose, glycerol-galactopyranoside and the amino acids proline (already increased in class 3), glycine and ornithine. Another cluster with metabolites that only decreased in needles in class 5 comprised organic acids (oxalic acid, glyceric acid, succinic acid and gluconic acid), fatty acids (myristic acid, hexanoic acid), starch and lactose and the amino acid aspartic acid.

**Figure 3:**
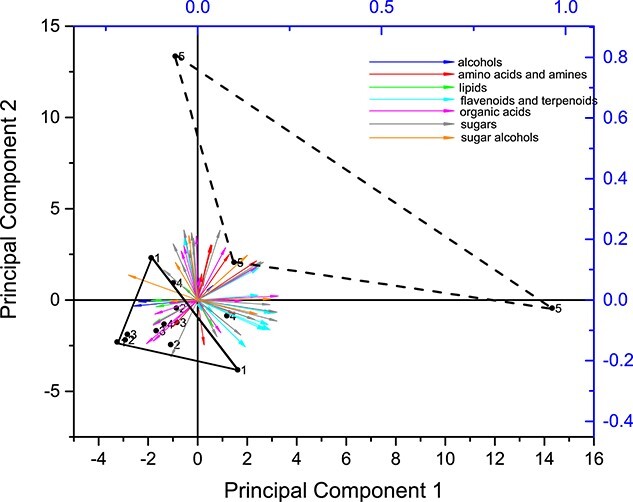
The PCA, including all detected metabolites in needles. Numbers 1–5 indicate the different defoliation classes. Each point represents one of the three replicate trees (with three technical replicates per tree) per defoliation class. The solid triangle with the solid line connects the trees from defoliation class 1 (0–22% defoliation), and the triangle with the dashed line connects the trees from class 5 (83–95% defoliation).

The results of the cluster analysis indicating that needles of defoliation class 5 (83–95% defoliation) showed a distinctly different metabolic profile compared with the other defoliation classes were corroborated by the PCA analysis (Figure 3). While the trees from classes 1 to 4 grouped closely together, class 5 separates and the plane spanning between the three replicates of class 5 did not overlap with any other class. The plane, which the three replicates of defoliation class 5 spans is considerably larger compared with the other defoliation classes indicating higher variability within this class. Principal component (PC) 1 explained 25.6% of the variance and PC 2 explained 23.5%. The two most important determinants for PC 1 were raffinose and abietic acid, and for PC 2, those were ribose and mannitol.

### Stress-related compounds in roots and needles

**Figure 4:**
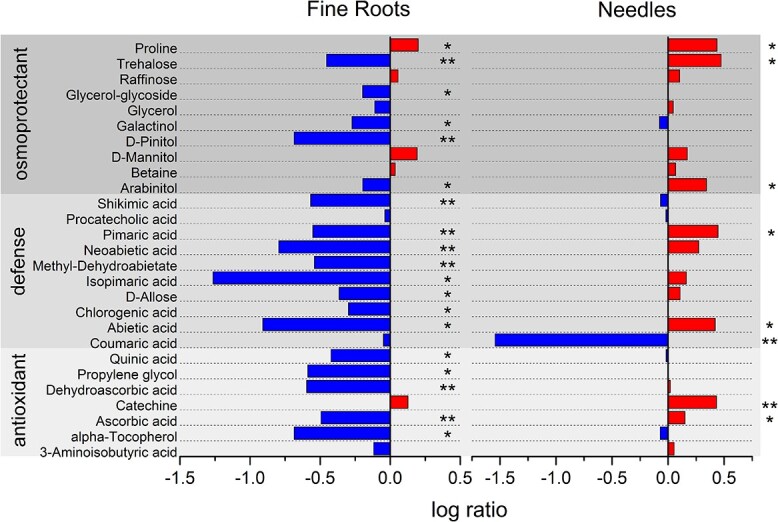
Log ratio changes in the major stress mitigating metabolites for roots and needles of Scots pine trees from defoliation classes 5 (83–95%) versus 1 (0–22%). Negative values indicate lower metabolite abundances and positive values higher abundances in highly defoliated trees (*n* = 3 with three technical replicates each). Shown are metabolites that differed significantly (*P* < 0.05) in either needles, roots or both tissues.

Overall stress-related (and potentially stress mitigating compounds) decreased in fine roots of strongly defoliated trees (as compared with trees with only 0–22% defoliation; for significance levels, see Table S3 available as Supplementary data at *Tree Physiology* Online), while the same compounds mostly increased in needles (Figure 4). A notable exception was coumaric acid, showing a stronger decrease in needles compared with roots (where there was no significant change; Table S3 available as Supplementary data at *Tree Physiology* Online). In roots, the osmoprotectants proline, raffinose, mannitol and betaine as well as catechin increased in strongly defoliated individuals of Scots pine. In needles, proline, trehalose, arabinitol, pimaric acid, abietic acid and catechin increased by a log ratio >0.3, indicating at least doubling of the concentration (for significance levels for needles, see Table S2 available as Supplementary data at *Tree Physiology* Online).

## Discussion

The main aim of our study was to understand the relationship between drought-affected tree vitality as indicated by defoliation and tree mortality risk on one hand and metabolic responses in leaves and needles on the other hand.

### Defoliation trajectories in Scots pine are early warning indicators for mortality

High needle loss of Scots pine tree crowns is a good predictor for mortality on our study site in Pfynwald. The mortality risk does not increase continuously between the different defoliation classes, but there is a tipping point at around 80–85% at which the mortality risk strongly rises. When surpassing this defoliation threshold, the physiological processes in a tree seem to be irreversibly disturbed so that recovery is barely possible anymore as the probability of dying within 6 years is 84% even if it still takes several years until mortality occurs. These results confirm the first part of the first hypothesis that increased defoliation would be a good predictor for mortality and are in line with previous studies. Hunziker et al. (2022) described a tipping point around 75% at Visp, a nearby Scots pine site also located in the Swiss Rhone valley. The temporal mortality patterns differ substantially between the two sites, as the site in Visp was affected by specific and severe mortality events, whereas mortality in Pfynwald occurred much more continually over time (Hunziker et al. 2022). This difference can be explained by the occurrence of more severe drought stress events in mid-to-late summer in Visp than in Pfynwald. Also, Scots pines died much faster in Visp (mostly within 2 years) than in Pfynwald after exceeding the defoliation threshold. Hence, the exact tipping point and the temporal development toward mortality may slightly vary depending on local conditions and previous stress exposure of trees, but Scots pines at different sites still show strong similarities in the relationship between defoliation and later mortality even when the site and stress legacy conditions differ substantially. Thus, our analysis of the metabolic profile across defoliation classes (from low to high needle loss) should be indicative for shifts in the commitment of metabolic pathways leading to changes in the metabolite composition on the trajectory to mortality.

### Only a few metabolites show a continuous increase in abundance with increasing defoliation and the most important one is proline

The second part of our first hypothesis was that in particular stress-related metabolites in needles should increase continuously with increasing defoliation. However, only few metabolites showed such a constant increase. Among them was proline, which is known to act as an osmoprotectant (Ghosh et al. 2022). Increased levels of free proline in plant tissues are typically seen in response to osmotic stress (Taylor 1996; Parida and Das 2005; Dluzniewska et al. 2007), and a strong relationship between osmotic stress tolerance and proline levels has been observed (Nanjo et al. 1999). The observed increase might, thus, point to the fact that increased defoliation is directly related to increasing drought stress and proline accumulation is a primary compensation mechanism. However, proline is a more general stress-mitigating metabolite as it acts as a membrane protectant, reactive oxygen scavenger, energy sink and signaling molecule (Szabados and Savouré 2010; Ghosh et al. 2022), and thus indicates response to various stressors. Thus, impacts of additional stressors such as heat, which often co-occurs with drought (Schuldt et al. 2020), might also play a role in the defoliation process. However, Jansen et al. (2014) showed that, in Douglas-fir needles, proline concentrations dropped under increased air temperature as proline is known to become toxic under heat stress (Rizhsky et al. 2004). Thus, the finding of increased proline abundance with defoliation rather indicates that heat is not a major driver of defoliation at our site even though heat is known to intensify drought stress (Allen et al. 2015). Together with proline, ornithine levels increased in needles in the defoliation classes 4 and 5, indicating that the metabolic pathway via ornithine δ-aminotransferase (EC 2.6.1.13) might play a major role for proline synthesis from ornithine (Majumdar et al. 2016). It is assumed that, both, the synthesis of proline from glutamate and via ornithine are activated under osmotic stress (Armengaud et al. 2004) but that the ornithine pathway plays also an important role under additionally limited nitrogen availability (Ghosh et al. 2022), which occurs often together with reduced water availability (Gessler et al. 2017). Needle nitrogen content was, however, not affected by defoliation in our study so that it is unlikely that, in the trees at our site, the increased synthesis of proline via ornithine was induced by nitrogen shortage. Closely linked to ornithine via the enzyme ornithine decarboxylase (EC 4.1.1.17) is putrescine, a precursor for the synthesis of other plant polyamines, which is, however, only increased in defoliation class 5. Polyamines might be an additional line of defense against drought-related damage as they are known to increase the antioxidant capacity, osmotic adjustment and control cellular water loss via regulating potassium channels (Chen et al. 2019).

### The overall metabolite profile in needles is kept rather constant over a wide range of defoliation but shows a tipping point at very high defoliation

Overall, we need to accept the alternative hypothesis for our first hypothesis. We see that stress-related metabolites increase at high defoliation; however, it is mostly not a continuous increase, but there seems to be a general metabolic tipping point between defoliation classes 4 and 5 and hence at ca 80–85% crown defoliation. This tipping point is indicated by the high Euclidean distance for class 5 in the cluster analysis (Figure 2) and by the fact that the plane spanning between the three replicates of defoliation class 5 in the PCA did not overlap with any other class, whereas all other classes showed overlaps (Figure 3). The metabolic tipping point is not limited to compounds that are involved in stress metabolism, as a clear difference between class 5 and the other classes also shows for metabolites that have more general functions such as sugars (e.g., fucose, which is involved in pathogen defense; Zhang et al. 2019; melibiose, turanose or glucose) and organic acids, some of which increased (citrate, malate and ascorbate, where the latter plays a central role as antioxidant; Xiao et al. 2021), whereas others decreased (coumaric acid, oxalate, glycerate and gluconic acid). The increase in sugars might also be related to the finding that drought stress can promote the conversion of glycosides into their corresponding aglycones to enhance the antioxidant effect (Cao et al. 2022), leading to the release of the sugar moieties of the glycosides. The increase in abundance of the tricarboxylic acid (TCA) cycle intermediates citrate and malate could indicate both higher and also lower activity of the TCA cycle as a central anaplerotic distribution hub. Generally, the expansion of malate and citrate pools could be a result of not only an inhibition of downstream metabolic pathways (in relation to the influx of metabolites into the TCA cycle) but also due to stimulated fluxes through the TCA cycle. It needs to be noted that the TCA ‘cycle’ is not a closed cycle during illumination (i.e., during the time when the needles were harvested in our study) (Tcherkez et al. 2009). Under illumination, the main supply pathway for the two branches of the open TCA ‘cycle’—one branch leading to malate and the other to citrate—is via phosphoenolpyruvate (PEP) and the enzyme PEP carboxylase (PEPC) (EC 4.1.1.31) (Werner et al. 2011). As it is known that PEPC activity is stimulated under stress conditions in another pine species (*Pinus halepensis*) (Fontaine et al. 2003), we might conclude that the increased metabolic flux toward the two TCA cycle branches led to the observed increases in citrate and malate abundance.

The observed metabolic homeostasis across a large range of defoliation, as seen here for the defoliation classes 1–4, was already described for soluble sugars (glucose, fructose and sucrose) and starch in Scots pine in long-term soil water manipulation treatments (Schönbeck et al. 2018). Our present observation indicates the high acclimation potential of Scots pine and points to the fact that even though needle area declines as a result of defoliation, the needle level metabolism is not strongly affected (but, only until a particular defoliation level is reached). We assume that the lower needle area compensates for reduced water availability (as the transpiring area is reduced), thus mitigating drought stress. The lower needle area may, however, also be a long-term adjustment of source activity (i.e., photosynthesis) to lower sink carbon demand under drought (in a sense as discussed by Körner 2015). Our results also show that there is a limit to such an adjustment as indicated by the strong shift in the metabolic profile when defoliation exceeds a threshold of ~80–85%. This is in line with our observations of the relationship between Scots pine defoliation class and mortality (Figure 1). The match between the defoliation tipping point that leads with a high probability to mortality and the metabolic tipping point might indicate that irreversible metabolic changes occur in needles of highly defoliated trees.

### High defoliation causes an increase in stress-related metabolites in needles but in contrast to a decrease in fine roots

A closer comparison of shifts in stress-related metabolite between the fine roots and the needles in highly defoliated trees (class 5) reveals clearly opposite patterns: while most compounds were increased in needles, there was a generally strong reduction in roots. In the roots, four (glycerol, galactinol, pinitol and arabinitol) out of five of polyols decreased and only mannitol increased, but only one of these compounds (galactinol) dropped in needles. Polyols are known to act primarily as osmoprotectants but also have antioxidant capacity and play a role in plant–pathogen resistance (Williamson et al. 2002). The reduction of polyol levels in roots is in line with the other defense compounds and antioxidants: shikimic acid, which is a central precursor for phenol synthesis and thus crucial for the defense against fungal pathogens (Witzell and Martín 2008) is strongly reduced in roots (and only slightly in needles). The decrease of resin acids such as isopimaric acid (impedes spore germination of pathogenic fungi), abietic acid (inhibits mycelia growth (Kopper et al. 2005), and pimaric and neoabietic acid (protect Scots pine against fungal attacks (Kaitera et al. 2021) indicates a reduction in the defense capacity against fungal infections in the roots. Ascorbate and dehydroascorbate are important constituents of the plant apoplast serving as a first-line antioxidative system (Haberer et al. 2007) and also regulating redox signaling pathways important for plant defense (Pignocchi and Foyer 2003). Tocopherol, instead, is lipid-soluble and is central for maintaining membrane integrity as it scavenges lipid peroxy radicals (Munné-Bosch and Alegre 2002) and hence prevents large-scale lipid peroxidation under biotic or abiotic stress. These antioxidative compounds are strongly decreased in the roots of heavily defoliated trees, while only tocopherol shows a slight reduction in needles, indicating an overall impairment or exhaustion of the defense against oxidative stress in the rooting system. The only stress-related compound more strongly reduced in needles than in roots is coumaric acid. Coumaric acid has been shown to be involved in defense against herbivorous insects (Usha Rani and Jyothsna 2010) and is also a precursor for the pathways of lignin biosynthesis (Chen et al. 2014). Thus, the decrease of this metabolite might indicate an increased susceptibility of needles against maturation feeding of pine shoot beetles (*Tomicus* spp.), which is widespread in our experimental area (Bose et al. 2022). As a consequence of such reduced defense capability, already strongly damaged trees might lose (at least parts of) their remaining needle area, potentially contributing to and accelerating mortality.

### The reduction of stress metabolites in roots of highly defoliated trees might indicate that the mortality risk is related to the inability to maintain root carbon supply

The second hypothesis supported by our observation that, in strongly defoliated Scots pine, most of the stress-related metabolites decreased in roots, whereas they (with the exception of coumaric acid) either increased or remained more or less constant in needles. This is in line with previous findings on whole-tree carbon allocation. For example, Huang et al. (2019) showed that the extreme carbon limitation imposed by reducing the atmospheric CO_2_ concentration led to a maintained allocation of new assimilates to aboveground tissue of Norway spruce at the expense of the supply to the roots. Under moderate carbon limitation, the same patterns were also observed for secondary defense metabolites such as phenolic compounds and monoterpenes. Comparable allocation patterns were observed for Scots pine at the site Pfynwald: low soil water supply led to reduced carbon allocation to roots and rhizosphere, while assimilates were retained aboveground (Joseph et al. 2020). Over the long term, the insufficient carbon investment belowground leads to the shrinkage of the tree-specific rhizosphere zone, resulting in reduced nitrogen and water uptake (Gao et al. 2021). Reduced water and nutrient supply in turn needs adjustment of the crown with a reduction of the needle area. With a strongly reduced needle area, trees are less and less able to provide sufficient assimilates for the demand of the roots, especially under dry conditions such as those occurring at the study site. Our results indicate that such carbon restriction to roots preceding mortality directly affects the major stress-related metabolites, making the rooting system especially susceptible to fungal pathogens and oxidative stress, which might accelerate dieback.

### Conclusion, limitations and outlook

Our results indicate that changes in stress-related needle metabolites may be a valuable predictor for an increased risk of mortality. However, the metabolic fingerprint seems not to be a better predictor than the estimate of the tree crown defoliation, which is far easier to generate. It rather provides a better understanding of the underlying mechanisms leading to mortality. In contrast to the mostly increasing stress-related metabolites in needles, our results suggest that the depletion of such metabolites in fine roots are a key element in the Scots pine mortality. The two key findings of our studies are (i) that the needle metabolic profile is kept constant (with proline, which acts as general stress mitigating metabolite, as main exception) across a wide range of defoliation and that strong changes only occur at a defoliation threshold, which is also indicative for an increased risk of mortality. (ii) When surpassing that defoliation threshold many compounds and especially the ones involved in defense against pests and pathogens, osmoprotection as well as antioxidants increase in abundance in needles. By contrast, these metabolites decrease in the fine roots. We thus conclude that the well-known reduction of carbon allocation to the rooting system under carbon limitation is directly reflected in the root metabolic fingerprint and there is thus reason to assume that a loss of metabolic functioning in the roots strongly contributes to the observed mortality trajectories.

As part of our study, we could only analyze the metabolic fingerprint in fine roots of trees with low (0–22%) and very high (83–95%) defoliation. The depletion of specific metabolites in fine roots might already occur at lower defoliation levels and could possibly indicate earlier than in needles if an increased crown defoliation of an individual tree is a sign either of temporary stress within the limits of resilience or of a progression on the trajectory toward mortality. Further studies focusing on the fine root metabolome could answer this relevant question.

A main limitation of the present study is the low number of three tree replicates (with three technical replicates each) per defoliation class for the needle samples and for defoliation classes 1 and 5 for the fine root samples. This is due to the fact that uprooting trees (an adequate method to get access to the fine roots of specific trees) requires a major logistic effort, is a substantial destructive intervention in nature and may have a substantial impact on a long-term monitoring area if too many trees are uprooted. Despite this shortcoming, the metabolic fingerprint along the defoliation class gradient detected in the present study is very consistent and does not indicate a sampling selection bias. Nevertheless, we would very much welcome further studies comparing the root and needle metabolic fingerprint in different defoliation classes at other sites and with other tree species to test if this is a species-specific reaction or a general pattern across species and genera.

## Authors’ contributions

A.G., M.H. and I.B. designed the experiment; A.G., S.H., T.N., M.K., M.H., C.H., L.S., A.K.B. and B.K. carried out the sampling, analysis and statistics; A.G. and S.H. wrote the manuscript with input from all other authors.

## Supplementary Material

Supplementary_material_final_R1_V2_tpae036

## Data Availability

All data will be made freely available by the authors.
