# Supplementary material for: The metabolic fingerprint of Scots pine—root and needle metabolites show different patterns in dying trees
Source: Tree Physiol. 2024 Mar 25;44(4):tpae036. doi: 10.1093/treephys/tpae036 (PMC11056600; doi:10.1093/treephys/tpae036)
Supplement: Supplementary_material_final_R1_V2_tpae036 [file supplementary_material_final_r1_v2_tpae036.docx]

## *S*upporting Information

Article title: **The metabolic fingerprint of Scots pine – root and needle metabolites show different patterns in dying trees**

Authors: Stefan Hunziker, Tatiana Nazarova, Michel Kather, Martin Hartmann, Ivano Brunner, Marcus Schaub, Andreas Rigling, Christian Hug, Leonie Schönbeck, Arun K. Bose, Bernd Kammerer, Arthur Gessler

The following Supporting Information is available for this article:

**Table S1** Additional leaf and growth traits of Scots pine for the five different defoliation classes. Data (with the exception of root and needle fresh weight/ dry weight ratio) were taken from Schönbeck et al. (2018). δ^13^C, phosphorus (P) and (N) content are from current year needles. Values shown are averages (n = 6 for current needle traits and shoot growth; n = 3 for needle fresh weight/ dry weight ratio; and n= 30-110 for DBH increment) ± SD. Significant differences among defoliation classes (p < 0.05; one way ANOVA with Tukey post-hoc test) if present are indicated by different upper-case letters. Relative DBH increment was determined over an eleven-year period, absolute shoot increment over a six-year period. Fresh/ dry weight ratios were determined for subsamples of the needle and root samples used for metabolite fingerprinting in the present study. Needles were weighed frozen (fresh weight) and then dried at 65° for four days and then weighed again. For other methodological details see Schönbeck et al. (2018). DBH: diameter at breast height; δ^13^C: carbon isotope composition referenced to Pee Dee Belemenite.

| **Defoliation class**  **Trait** | **1** | **2** | **3** | **4** | **5** |
| --- | --- | --- | --- | --- | --- |
| **δ^13^C (‰)** | -27.7 ± 0.4 | -28.2 ± 0.5 | -27.5 ± 0.5 | -27.5 ± 0.3 | -27.2 ± 0.4 |
| **Nitrogen (%)** | 1.47 ± 0.15 | 1.45 ± 0.16 | 1.39 ± 0.11 | 1.30 ± 0.14 | 1.31 ± 0.11 |
| **Phosporus (mg kg^-1^)** | 698 ± 44 AB | 680 ± 45 AB | 667 ± 37 B | 608 ± 50 B | 758 ± 40 A |
| **Needle fresh weight/ dry weight ratio** | 2.17 ± 0.15 | 2.19 ± 0.13 | 2.17 ± 0.11 | 2.16 ± 0.16 | 2.17± 0.13 |
| **Needle fresh weight/ dry weight ratio** | 1.92 ± 0.11 | 1.94 ± 0.09 | 1.90 ± 0.12 | 1.91 ± 0.14 | 1.92± 0.11 |
| **Relative DBH increment (%)** | 15.8 ± 1.3 A | 11.3 ± 1.1 B | 6.1 ± 0.7 C | 2.6 ± 0.7 D | 1.4 ± 0.8 D |
| **Shoot increment (cm)** | 20.7 ± 2.9 A | 20.2 ± 4.9 AB | 19.9 ± 3.4 A | 13.7 ± 2.2 BC | 12.4 ± 1.2 C |

**Table S2** Statistical differences for metabolites in needles between defoliation class 1 (0-22% needle loss) and the other four defoliation classes. ***: p<0.001, **:0.001< p <0.010, *: 0.010 < p <0.050, NS: p >0.051, and the ↑ and ↓ indicate a positive and a negative effect, respectively.

| **Metabolite** | **Class 1 *Vs* 2** | **Class 1 *Vs* 3** | **Class 1 *Vs* 4** | **Class 1 *Vs* 5** |
| --- | --- | --- | --- | --- |
| 2_3 Butanediol | NS | NS | NS | NS |
| 2O-Glycerol-Alpha-D-Galactopyranoside | NS | NS | NS | NS |
| 3- Aminoisobutyric acid | NS | NS | NS | NS |
| 4-Coumaric acid | NS | NS | NS | **↓ |
| Abietic acid | NS | NS | NS | *↑ |
| Alpha tocopherol | NS | NS | NS | NS |
| Alpha-D-Arabinopyranose | NS | NS | NS | NS |
| Lactose | NS | NS | NS | **↑ |
| Alpha-ketuglutarate | NS | NS | NS | NS |
| Alpha-Lactose | NS | NS | NS | NS |
| Arabinitol | NS | NS | NS | *↑ |
| Ascorbic acid | NS | NS | * | *↑ |
| Aspartic acid | NS | NS | NS | *↑ |
| Beta-alanine | NS | NS | NS | NS |
| Betaine | NS | NS | NS | NS |
| Caryophyllene | NS | NS | NS | NS |
| Catechine | NS | NS | NS | **↑ |
| Citric acid | NS | NS | NS | **↑ |
| D-Allose | NS | NS | NS | NS |
| D-Fructopyranose | NS | NS | NS | NS |
| D-Fructose | NS | NS | NS | NS |
| D-Gluconic acid | NS | NS | NS | NS |
| D-Glucose | NS | NS | NS | NS |
| D-Lactic acid | NS | NS | NS | NS |
| D-Malic acid | NS | NS | NS | NS |
| D-Mannitol | NS | NS | NS | NS |
| D-Mannose | NS | NS | NS | NS |
| D-Pinitol | NS | NS | NS | NS |
| D-Sphingosine | NS | NS | NS | NS |
| D-Threitol | NS | NS | NS | *↑ |
| Dehydroascorbic acid | NS | NS | NS | NS |
| Epigallocatechin | NS | NS | NS | NS |
| Galactinol | NS | NS | NS | NS |
| Gemacrene A | NS | NS | NS | NS |
| Glyceric acid | NS | NS | NS | *↓ |
| Glycerol | NS | NS | NS | NS |
| Glycerol-glycoside | NS | NS | NS | NS |
| Glycine | NS | NS | NS | *↑ |
| Hexanoic acid | NS | NS | NS | NS |
| Humulene | NS | NS | NS | NS |
| Isopimaric acid | NS | NS | NS | NS |
| L-Alanine | NS | NS | NS | NS |
| L-Fucose | NS | NS | NS | *↑ |
| L-Glutamic acid | NS | NS | NS | NS |
| L-Ornithine | NS | NS | NS | **↑ |
| Lactose | NS | NS | NS | NS |
| Melibiose | NS | NS | NS | NS |
| Myo-Inositol | NS | NS | NS | NS |
| Myristic acid | NS | NS | NS | NS |
| Neoabietic acid | NS | NS | NS | NS |
| Nonanoic acid | NS | NS | NS | NS |
| Oxalic acid | NS | NS | NS | *↓ |
| Palmitic acid | NS | NS | NS | NS |
| Pimaric acid | NS | NS | NS | *↑ |
| Procatecholic acid | NS | NS | NS | NS |
| Proline | NS | NS | NS | *↑ |
| Putrescine | NS | NS | NS | **↑ |
| Quinic acid | NS | NS | NS | NS |
| Raffinose | NS | NS | NS | NS |
| Ribonic acid | NS | NS | NS | NS |
| Ribose | NS | NS | NS | NS |
| Shikimic acid | NS | NS | NS | NS |
| Stearic acid | NS | NS | NS | NS |
| Starch | NS | NS | NS | *↓ |
| Succinic acid | NS | NS | NS | NS |
| Sucrose | NS | NS | NS | **↓ |
| Threonic acid | NS | NS | NS | NS |
| Trehalose | NS | NS | NS | *↑ |
| Turanose | NS | NS | NS | ***↑ |

**Table S3** Statistical differences for metabolites in roots between defoliation class 1 (0-22% needle loss) defoliation class 5. ***: p<0.001, **:0.001< p <0.010, *: 0.010 < p <0.050, NS: p >0.051, and the ↑ and ↓ indicate a positive and a negative effect, respectively.

| **Metabolite** | **Class 1 *Vs* 5** |
| --- | --- |
| 3- Aminoisobutyric acid | NS |
| Abietic acid | *↓ |
| Alpha tocopherol | *↓ |
| Arabinitol | *↓ |
| Ascorbic acid | **↓ |
| Betaine | NS |
| Catechine | NS |
| Chlorogenic acid | *↓ |
| Coumaric acid | NS |
| D-Allose | *↓ |
| D-Mannitol | NS |
| D-Pinitol | **↓ |
| Dehydroascorbic acid | **↓ |
| Galactinol | *↓ |
| Glycerol | NS |
| Glycerol-glycoside | *↓ |
| Isopimaric acid | **↓ |
| Methyl-dehydroabietate | **↓ |
| Neoabietic acid | **↓ |
| Pimaric acid | **↓ |
| Procatecholic acid | NS |
| Proline | *↑ |
| Propylene glycol | *↓ |
| Quinic acid | *↓ |
| Raffinose | NS |
| Shikimic acid | **↓ |
| Trehalose | **↓ |

**References**

Schönbeck L, Gessler A, Hoch G, McDowell NG, Rigling A, Schaub M, Li M-H. 2018. Homeostatic levels of nonstructural carbohydrates after 13 yr of drought and irrigation in *Pinus sylvestris*. New Phytologist 219: 1314-1324.
